# Peony and licorice decoction fumigation treatment for strephenopodia after stroke: Study protocol for a randomized controlled pilot trial

**DOI:** 10.1097/MD.0000000000023600

**Published:** 2020-12-11

**Authors:** Chengyang Jing, Li Zhou, Juanjuan Ai, Zongheng Li, Jiabao Wu, Yiting Sun, Shuang Zhao

**Affiliations:** a Department of Rehabilitation; b Department of Emergency; c Department of Education, Dongzhimen Hospital Affiliated to Beijing University of Chinese Medicine; d First Clinical Medical School, Beijing University of Chinese Medicine, Beijing, China.

**Keywords:** biomechanical mechanisms, fumigation therapy, peony and licorice decoction, randomized controlled trial, strephenopodia

## Abstract

**Background:**

As one of the most common functional disabilities in stroke patients with hemiplegia, poststroke strephenopodia (PSS) seriously affects the life quality of patients, and causes mental and emotional disorders. Some studies have suggested that the traditional Chinese medicine fumigation therapy could be an effective intervention method for patients with PSS. This study aims to investigate the biomechanical effect of the classic prescription peony and licorice decoction (PLD) fumigation treatment for PSS.

**Methods/Design:**

This study is a multicenter, randomized, placebo-controlled, double blind trial. A total of 190 patients with PSS according to the inclusion criteria will be recruited in 3 centers and randomly distributed to either the intervention group or the control group in a 1:1 ratio. The intervention group will receive PLD fumigation treatment, while the control group will receive placebo fumigation treatment. All patients will receive standardized modern rehabilitation treatment according to the “Chinese Guidelines for Stroke Rehabilitation” (2011 version). The primary outcome measure is medial plantar area (Metatarsal 1+ Metatarsal 2 + Heel Medial) generating from the RSSCAN gait system. The secondary outcome measures contain the scores of clinical scales including Berg Balance Scale, Fugl-Meyer Assessment, Modified Ashworth Scale, Barthel Index, and Stroke-Specific Quality of Life Scale. All assessments will be implemented at baseline, 4 weeks after intervention and at the end of 3 months’ follow-up. Intention-to-treat analysis and per-protocol analysis will be applied in this trial.

**Discussion:**

The results of this study are expected to verify the clinical effect of PLD fumigation treatment for strephenopodia after stroke, and to explore the related biomechanical mechanisms by objective evaluation parameter.

**Trial registration:**

Chinese Clinical Trial Registry, ChiCTR2000032433. Registered on 28 April 2020. http://www.chictr.org.cn/showprojen.aspx?proj=52644

## 1 Introduction

Strokes are a type of cerebrovascular disease characterized by high incidence, high mortality, and high disability. The authoritative survey showed that strokes had become the leading cause of disability and the second leading cause of death in the world.^[1]^ A systematic review of stroke confirmed that stroke incidence in China had increased over the last decade.^[2]^ With the fast development of modern medicine, most stroke patients can be treated in time and the mortality rate of strokes can be controlled to a certain extent.^[3]^ However, long-term disability is highly prevalent in poststroke patients and the disability rate is even more than 40 percent.^[4]^ Poststroke strephenopodia (PSS) is one of the most common functional disabilities in stroke patients with hemiplegia.^[5]^ Studies showed that the incidence of PSS ranged from 17% to 38% in the population of patients with strokes, and 4% to 9% of stroke survivors were being disabled.^[6]^ The life quality of stroke patients is seriously affected by the abnormal gait,^[7]^ balance disorder,^[8]^ life restriction, and the consequent abnormal mental mood ^[9,10]^ which are caused by PSS.

Modern rehabilitation techniques such as foot support fixation, plantar inhibition, and other good limb placement methods are used in the acute phase of stroke to prevent the occurrence of PSS.^[11]^ To some extent, these methods reduce the incidence of PSS in stroke patients. However, due to the relatively serious condition of patients in the acute stage, most treatment schemes focus on the intervention of vital signs.^[12]^ The intervention of early rehabilitation treatment is often neglected. As a result, most stroke patients received rehabilitation treatment only when their vital signs were relatively stable.^[13]^ For the spasmodic strephenopodia forming during the recovery period, rehabilitation techniques, such as passive joint activity training and weight loss gait training, are mostly adopted to relieve muscle spasm. For the more seriously refractory strephenopodia, even the Botox injection or surgery is used to inhibit the excessive flexion spasm of the medial muscles.^[14,15]^ However, the outcome of the above therapies is not satisfactory. Such situations drive us to seek a more effective method for the treatment of PSS.

The traditional Chinese herbal medicine is used to fumigate the patient's diseased area by the gas, which is generated by the boiling of drugs, to achieve the therapeutic effect. Absorption through the skin plays a role in avoiding the stimulation of drugs to the gastrointestinal tract, reducing the burden for the liver and kidney, and making the incidence of adverse drug reactions being significantly reduced.^[16]^ For the patients who are not suitable for oral administration of drugs, it is undoubtedly a good way to administer drugs. From Zhongjing Zhang's Treatise on Febrile Diseases, the classic prescription peony and licorice decoction (PLD), known as “traditional Chinese medicine morphine,” is primarily used to treat visceral pain, painful muscle spasms, menstrual pain, and so on.^[17,18]^ Modern research confirmed that total paeoniflorin in white peony and total glycyrrhizin in licorice have strong anti-inflammatory and analgesic effects.^[19]^ The oral treatment of PLD can significantly improve limb motor function and activities of daily living for patients with spastic hemiplegia after stroke.^[20]^ However, for the treatment of PSS, the clinical application of PLD is carried out mostly by oral administration and few studies focus on the fumigation and steaming therapy of PLD. Furthermore, it is a pity that the mechanism of PLD fumigation treatment is still unclear.

This study has been designed to verify the clinical effect of PLD fumigation treatment for strephenopodia after stroke, and to explore the related biomechanical mechanisms by objective evaluation parameter from the RSSCAN gait system.

## 2 Methods/design

### 2.1 Study design

**Figure 1:**
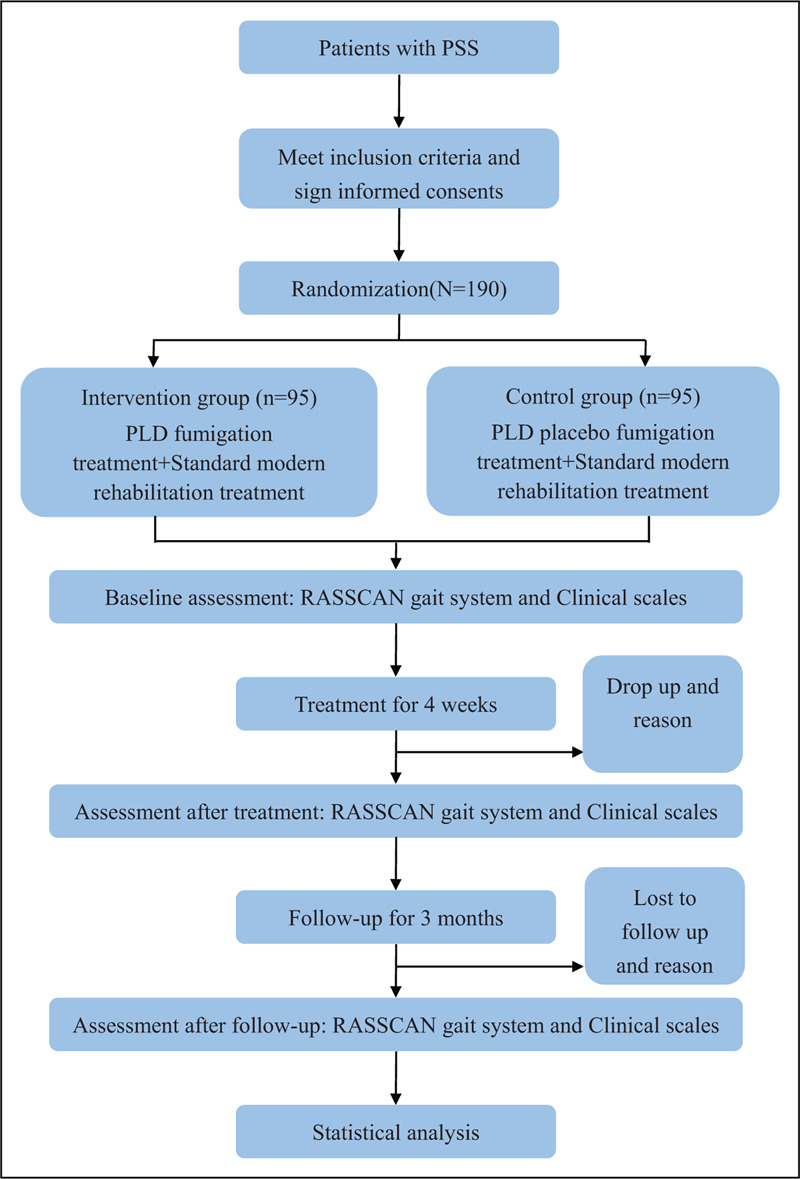
Flow chart of study design.

This is a multicenter, randomized, placebo-controlled, double blind trial. A total of 190 patients meeting the inclusive criteria will be recruited and then randomly allocated into 2 groups in a 1:1 ratio using SPSS 25.0 (IBM, USA) for Windows (Chicago, IL, USA). Both groups will receive standard modern rehabilitation treatment according to the “Chinese Guidelines for Stroke Rehabilitation” (2011 version).^[21]^ Patients will stick to the treatment they previously have had, and will be given present general treatment when acute exacerbation of stroke occurs during the trial. The intervention group will receive PLD fumigation treatment, while the control group will receive placebo fumigation treatment. The treatments will be taken once a day lasting 30 minutes, 5 days per week. An objective biomechanical parameter, the medial plantar area (metatarsal 1+ metatarsal 2 + heel medial) from the RSSCAN gait system, will be used as the primary measure to assess the outcome. Scores of Berg Balance Scale (BBS), Fugl-Meyer assessment, Modified Ashworth Scale, Barthel Index, and Stroke-Specific Quality of Life Scale (SSQOL) will be used as the secondary measure to assess the outcome. All assessments will be conducted at baseline, a 4-week treatment and a 3-month follow-up. All participants will provide signed informed consent before proceeding with the trial. The flow chart of this trial is summarized in Fig. 1. The study timeline and event schedule are set up according to the Standard Protocol Items: Recommendations for Interventional Trials (SPIRIT) 2013 Statement (Additional file 1), as detailed in Table 1.^[22]^

**Table 1:** Timing of treatment visits and data collection.

	Study period			
	Enrollment	Baseline	Treatment phase	Follow-up phase
Time point	−1 wk	0 wk	4 wks	12 wks
Enrollment				
Eligibility screen	×			
Informed consent	×			
Demographic information	×			
Stroke type	×			
Medical history	×			
Disease history	×			
Randomization	×			
Intervention				
PLD fumigation treatment		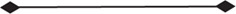		
PLD placebo fumigation treatment		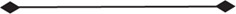		
Standard modern rehabilitation treatment		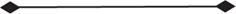		
Primary outcomes				
Medial planter area (M1+M2+HM)		×	×	×
Secondary outcomes				
BBS		×	×	×
FMA		×	×	×
MAS		×	×	×
BI		×	×	×
SSQOL		×	×	×
Safety				
Adverse events		×	×	×
Success of blinding			×	

BBS = Berg Balance Scale, BI = Barthel Index, FMA = Fugl-Meyer Assessment, HM = Heel Medial, M1 = Metatarsal 1, M2 = Metatarsal 2, MAS = Modified Ashworth Scale, PLD = peony and licorice decoction, SSQOL = Stroke-Specific Quality of Life Scale.

### 2.2 Ethical issues

Participants and Fugal their families will be fully informed of the details about this study before the patients take part in this research. Informed consent includes probable risks, potential benefits, as well as the obligations as stated in the Declaration of Helsinki 2013. Meanwhile, participants will also be told that the participation in the trial is entirely voluntary and they can withdraw from this study at any time for any reason. All recruited participants will be provided written informed consent before they take part in this study. The protocol has been registered with the Chinese Clinical Trial Registry: ChiMCTR2000003253. Ethical Committee of Dongzhimen Hospital Affiliated to Beijing University of Chinese Medicine has approved the study protocol with identifier DZMEC-KY-2019-200. In case of any changes to the study protocol, a written application will be submitted to the ethical committee. On that basis, the ethical committee will decide whether it is necessary or not to change the protocol.

### 2.3 Participant recruitment

This multicenter randomized controlled pilot trial will be conducted at 3 trial sites in Beijing in mainland China:

(1) Dongzhimen Hospital Affiliated to Beijing University of Chinese Medicine,
(2) Dongfang Hospital Affiliated to Beijing University of Chinese Medicine, and
(3) The Third Affiliated Hospital of Beijing University of Chinese Medicine.

Patients meeting inclusion criteria will be recruited through posters in the inpatient or outpatient departments. Prospective participants will be invited into this study after presenting the signed informed consent. The recruitment will begin in June 2020 and will continue until 190 patients are enrolled.

### 2.4 Inclusion criteria

- Confirmed stroke patients with results from computed tomography or magnetic resonance imaging
- Aged between 35 and 75, male or female
- First episode of stroke or with a history of stroke but with no serious neurofunctional disability and modified Ranking Scal grade ≤2
- Stable condition after stroke and within 6 months of the duration
- With PSS and be able to walk at least 6 m
- Blood pressure lower than 160/90 mm Hg
- Sufficient cognition to follow commands and Mini-Mental State Examination (MMSE) score >24
- Never used fumigation treatment before
- The patients or their legal guardians sign the informed consents

### 2.5 Exclusion criteria

- Received surgery or thrombolytic therapy
- Duration of stroke is more than 6 months
- Stroke without PSS, or with PSS but could not walk 6 meters
- Vital signs are not stable or with worsening conditions such as new infarction or bleeding
- Combined with other cerebral diseases such as subarachnoid hemorrhage, cerebral hemorrhage, brain tumor, brain trauma, and so on
- Combined with lumbar vertebrae disease, knee joint disease, foot disease, and other diseases that can affect the patient's walking gait
- Combined with severe dysfunction of heart, lung, liver, kidney, and blood system
- Combined with moderate to severe cognitive comprehension or visual impairment that can affect rehabilitation treatment or gait examination
- Pregnant or lactating women
- Participating in other clinical trials.

### 2.6 Sample size

The sample size calculation was based on the medial plantar area. According to previous studies,^[23]^ assuming the medial plantar area is 20.15 in the intervention group and 18.94 in the control group; therefore, the mean difference between 2 groups is 1.21 with standard deviations of 2.12 and 2.46. With a type I error of 5% (α = 0.05) and 90% power (β = 0.10), the estimated required sample size is 76 participants per group based on the formula:

$$n=2\left[{\left(\mu_{\alpha }+\mu_{\beta }\right)}^{2}\sigma^{2}\right]/\delta^{2}$$

Considering a 20% dropout rate during the study, 95 patients will be enrolled in each group and the total sample size will be 190.

### 2.7 Randomization and allocation concealment

The stratified block randomization scheme will be applied in this trial. An independent statistician will generate the random allocation sequence using SPSS 25. 0 (IBM, USA). All participants who meet the inclusion criteria will be randomly assigned to an intervention group or control group (95 cases each) at a 1:1 ratio by the computer-generated random sequences. Opaque envelopes will be used to seal generated sequences and submitted to 3 centers. The allocation of eligible participants will be concealed from their caregivers and therapists. The therapists will only take charge of the allocated treatments for patients. Moreover, the allocation will also be concealed to the outcome assessors and data statistical analysts.

### 2.8 Blinding

In this study, the double-blind method will be implemented. A “third party” staff that is trained and does not participate in the experiment will manage and supervise the performance of the blinding method. Firstly, the random computer-generated assignments will be sealed in opaque envelopes. The participants will only be told that they will be randomly allocated to either intervention group or control group, and both be treated with regular rehabilitation therapies. And the researchers including therapists, assessors, statisticians, and data analysts will be blinded to the group allocation. All of them will work independently and separately. Secondly, the placebo used in control group will be made of 5% PLD and 95% dextrin, which will ensure it mimics the appearance and smell of PLD.

All researchers will be trained before the trial to ensure the successful implementation of the blinding method. Unblind will also be considered if adverse events occur or the trial ends.

### 2.9 Interventions

The patients will receive fumigation treatment (20 minutes) and standardized modern rehabilitation treatment (30 minutes) every day, once a day, 5 days per week (from Monday to Friday) across 4 weeks.

The intervention group will receive PLD fumigation treatment, while the control group will receive placebo fumigation treatment. An expert panel including 3 qualified therapists from the rehabilitation department and 3 senior doctors from the neurology department will set up the fumigation treatment program. According to the proportion of ancient prescription at 1:1, the main components of PLD are shown in Table 2. All Chinese herbal medicine will be made into granules in advance. Each bag of granules for fumigation treatment contains 240 g. The only difference is that each bag of placebo contains 5% PLD only and 95% dextrin. The components of the PLD granules are produced and packed by Bei Jing Kang Ren Tang Pharmaceutical Co. Ltd. The clinical research coordinators will inspect and ensure that all granules meet required quality standards before the trial. When each fumigation treatment begins, the therapist will obtain one bag of granules from the clinical research coordinator, then mix the granules and 400 ml of boiling water into the fumigation treatment machine (HB3000, Suzhou Hao Bo Medical Equipment Co. Ltd, Jiangsu Province, Taicang City). The medial knee and ankle joints of the affected side will be selected as fumigation sites to relieve the spasm pain of the medial muscle group and the strain pain of the lateral muscle group. Patients need to receive the fumigation treatment under the guidance of the therapists who are responsible for them. Any other fumigation treatment is prohibited during the treatment and follow-up period.

**Table 2:** Main components of penony and licorice decoction in fumigation treatment.

Chinese name	Latin name	Amount (g)
Chinese herbal formula penony and licorice decoction		
Bai Shao	Radix Paeoniae Alba	120
Gan Cao	Radix Glycyrrhizae	120

All patients in 2 groups will receive the same standardized modern rehabilitation treatment according to the “Chinese Guidelines for Stroke Rehabilitation” (2011 version). The main content of modern rehabilitation techniques is Bobath method and proprioceptive neurodevelopmental facilitation technique, which includes good limb position, muscle strength and joint activity training, knee-ankle joint control training, weight loss gait training, and so on. Five qualified and experienced rehabilitation specialists in 3 trial sites will select and conduct appropriate treatment programs according to the participants’ symptoms. All of them will receive the unified training before the start of the trial. Meanwhile, the patients will stick to the treatment they previously had, and will be given present general treatment if acute exacerbation of stroke occurs during the trial.

### 2.10 Follow-up

After finishing the 4-week treatment, all patients will enter the 3-month follow-up period. In view of the particularity of stroke rehabilitation and the ethical factors that need to be considered, patients can receive other possible salutary rehabilitation treatment except for the prohibition of additional fumigation treatment. During the 3-month follow-up period, patients will be required to fill out a form to record their specific recovery process during this period. At the end of the follow-up, all forms will be returned to the researchers for evaluation.

### 2.11 Outcome

#### 2.11.1 Primary outcome

In this trial, the data of medial plantar area (metatarsal 1+ metatarsal 2 + heel medial) generating from the RSSCAN gait system (RSSCAN International, Olen, Belgium) will be selected as primary outcome. The RSSCAN gait system consists of a pressure test plate with sensors arrayed, a data collector, and data acquisition software. The pressure test plate (2 m × 0.4 m, 16,384 sensors, 100 Hz) will be laid in the middle of the plastic runway, and the thickness of the runway is the same as the plate. The patients will be told to walk at their normal comfortable pace from one end of the plate to the other end with a natural gait. For each test, the patients will practice walking 3 times on the plate before beginning the formal test. The pressure test plate is directly connected to the data acquisition software through the data collector.

The criteria for the validity of the test data are as follows: the computer transmission system shows complete footprints; during the test, the participants look straight ahead and walk naturally without deliberately treading; there is no obvious gait change on the plate. The RSSCAN gait system in each center is the same model for ensuring the homogeneity of the data. Three qualified RSSCAN system operators who had received standardized training before the trial will accomplish all tests in their respective centers.

#### 2.11.2 Secondary outcome

The scores of clinical scales, including BBS, Fugl-Meyer Assessment, Modified Ashworth Scale, Barthel Index, and Stroke-Specific Quality of Life Scale, will be used as secondary outcome.

##### 2.11.2.1 Berg Balance Scale

As the most widely used balance evaluation scale for stroke patients in the world, the BBS will be used to assess the patients’ balance ability under static and dynamic conditions. Total score of the BBS is 56. The higher the score, the better the balance ability of patients.

##### 2.11.2.2 Fugl-Meyer assessment

The Fugl-Meyer assessment will be applied to assess the motor function level of patients. Since this study focuses on the test of lower limbs, we will only select part of the lower limb evaluation with a total score of 34. This assessment will evaluate in detail the motor function and reflex activity of affected lower limb joints including ankle joint and knee joint.

##### 2.11.2.3 Modified Ashworth Scale

The Modified Ashworth Scale is a simple grading system that scores from 0 (normal) to 4 (severe), which will be used to evaluate the level of muscular tension of the patients briefly.

##### 2.11.2.4 Barthel Index

The Barthel Index contains 10 basic daily activities and its total score is 100, which will be used to assess the daily living ability of patients by the score.

##### 2.11.2.5 Stroke-specific Quality of Life Scale

The SSQOL consists of 12 aspects and 78 entries including energy, family roles, language, mobility, mood, and so on. The SSQOL will be applied to fully evaluate the quality of patients’ activities regarding daily living. The higher the score, the better the quality of patients’ activities in carrying out daily living functions.

### 2.12 Safety assessment

Safety assessment will be used to avoid adverse events. Any adverse events that occur during the intervention period, such as local redness or pain, local scalds or itching, and dizziness, will be recorded and reported to the chief researcher and ethics committees of 3 centers. They will analyze the causality with fumigation treatment and determine whether to unblind according to the patient's condition. In the case of stroke recurrence or other worsening conditions, the patient will withdraw from the study and receive further treatment for free.

### 2.13 Data management and monitoring

Before this study, the Data Safety and Monitoring Committee (DSMC), composed of experts in rehabilitation, neurology, ethics, and statistics, will be set up for data management and monitoring. The committee is independent from trial investigators, and has no competing interests. All the researchers involved in data management will be trained. Firstly, 3 assessors will be responsible for acquisition and assessment of patients’ information during the study. After assessors finish the case report forms completely, 2 data collectors will validate the completeness and consistency of the data, and then convert the credible paper data to electronic data. All paper and electronic data related to the study will be safely kept in the Clinical Research Center of Beijing Dongzhimen Hospital. Only the independent statisticians will have access to the final complete data, others who have any questions will be required written requests to the DSMC to get permission.

The DSMC is also in charge of monitoring. Members of the committee will monitor the overall quality and completeness of the data, interview assessors, examine original documents, and make sure that the study is implemented with the principles of this protocol. In case of any changes to the study protocol, the DSMC will submit the written application to the REC to obtain permission. In addition, the monitors will verify that all adverse events will be recorded in the correct format. The DSMC will audit the study through regular interviews and the periodic review will be done every 2 months.

### 2.14 Statistical analysis

Statisticians who are independent from the trial will be responsible for the statistical analysis. The SPSS 25.0 (IBM, USA) for Windows (Chicago, IL, USA) will be used. Categorical variables will be presented with frequencies or percentages and continuous variables will be presented as the mean and standard deviation. The analysis will mainly compare efficacy between the intervention group and the control group, including primary and secondary outcomes. Changes in all outcome measurements of before and after the treatment and of the between group will be analyzed. The demographic and clinical characteristics of the 2 groups will be compared at baseline applying unpaired 2 sample *t*-tests (continuous data) and Chi-square analysis (categorical data). Rank sum test will be used when the normal distribution hypothesis is not met. Considering some participants may fail follow-up, we will conduct both intention-to-treat analysis and per-protocol analysis. The intention-to-treat analysis will include all the participants. The missing data will be treated by multiple imputations. The per-protocol analysis will incorporate the participants who follow all the time points outcome measurement and fully comply with the treatment schedule in the intervention group. The statistical significance threshold will be set at 0.05 (2-sided), with 95% confidence intervals.

### 2.15 Quality control

An independent quality control team will be set up for training 4 clinical research coordinators who are responsible for enrolling patients, acquiring informed consent, and requesting randomization. The training contents include patient recruitment, disease assessment, and data collection, which will minimize selection bias. In the process of data collection, patients’ combined medication and nonintervention treatment will be strictly noted, and the influence of the above-mentioned interfering factors will be excluded in the statistical analysis. The quality control team will also be answerable for the long-term communication and health education of patients. The above measures will help to improve compliance of patients and reduce the lost rate of follow-up.

## 3 Discussion

Although PSS seriously affects the life quality of stroke patients during the recovery periods, effective treatment is still lacking in clinics. At present, numerous domestic and foreign scholars think that exercise therapy is the basic treatment for PSS, however, the actual effect is less than expected. Oral or intrathecal injection of baclofen is also a common clinical method.^[24,25]^ Its effect is relatively significant, but it will also have an impact on normal muscle strength, which is not conducive to rehabilitation training. Therefore, seeking an effective treatment with few side effects appears to be particularly important. In China, traditional Chinese medicine fumigation therapy is widely used in clinics because of its characteristics of external treatment and direct action to the disease location. Previous study had shown that the application of traditional Chinese medicine fumigation therapy in the rehabilitation of PSS had a good theoretical basis and certain therapeutic advantages.^[26]^ However, there are few current studies on the application of PLD fumigation treatment of PSS, and the curative effect needs to be supported by the evidence of clinical trials. This study was designed to determine its real efficacy.

To achieve the best performance in the field, the RSSCAN gait system used to be designed for providing accurate and objective biomechanical parameters to formulate and improve athletes’ gait.^[27]^ Surprisingly, researches revealed that the application of RSSCAN gait system in clinical studies of diabetes, multiple sclerosis, and knee osteoarthritis also achieved satisfactory results by providing quantitative assessments.^[28]^ Based on it, we hope to determine the real effect of PLD fumigation treatment of PSS through the objective biomechanical parameters of the system. At the same time, the changes of parameters before and after treatment may also explain the biomechanical mechanisms under its curative effect.

Some limitations are inevitable in this study. Firstly, despite assessor-blinding, several patients who had been fumigated with traditional Chinese medicine will likely know which group they belong to according to the smell of the steam. Thus, we will select patients who have never received traditional Chinese medicine fumigation treatment before and keep patients separate from each other. Secondly, as this study is intended as a pilot study for further larger clinical studies, sample size is another limitation.

The results of this study are expected to verify the clinical effect of PLD fumigation treatment for strephenopodia after stroke, and to explore the related biomechanical mechanisms by objective evaluation parameter. The detailed interpretations of data in the trial will provide foundations for future larger clinical studies.

## Author contributions

**Conceptualization:** Juanjuan Ai, Zongheng Li.

**Data curation:** Jiabao Wu, Yiting Sun.

**Funding acquisition:** Juanjuan Ai.

**Methodology:** Zongheng Li.

**Project administration:** Juanjuan Ai.

**Software:** Jiabao Wu, Yiting Sun.

**Visualization:** Shuang Zhao.

**Writing – original draft:** Chengyang Jing, Li Zhou.

**Writing – review & editing:** Chengyang Jing.
